# Metabolomic and Confocal Laser Scanning Microscopy (CLSM) Analyses Reveal the Important Function of Flavonoids in *Amygdalus pedunculata* Pall Leaves With Temporal Changes

**DOI:** 10.3389/fpls.2021.648277

**Published:** 2021-05-19

**Authors:** Yueyue He, Lei Pan, Tao Yang, Wei Wang, Cong Li, Bang Chen, Yehua Shen

**Affiliations:** ^1^Key Laboratory of Synthetic and Natural Functional Molecule Chemistry of Ministry of Education, College of Chemistry and Materials Science, National Demonstration Center for Experimental Chemistry Education, Northwest University, Xi’an, China; ^2^Shaanxi Academy of Forestry, Xi’an, China; ^3^Technology Research Center of Amygdalus pedunculata of State Forestry and Grassland Administration, Yulin, China; ^4^Key Laboratory of Silviculture of the State Forestry Administration, The Institute of Forestry, The Chinese Academy of Forestry, Beijing, China

**Keywords:** *Amygdalus pedunculata* Pall leaves, metabolomics, flavonoids, UPLC–QTOF–MS, confocal laser scanning microscopy

## Abstract

*Amygdalus pedunculata* Pall [Rosaceae, Prunus, *Prunus pedunculata* (Pall.) Maxim.] belongs to the Rosaceae family and is resistant to cold and drought. Ultra-performance liquid chromatography quadrupole time-of-flight mass spectrometry and metabolomics were used to track the changes in bioactive metabolites during several stages of *Amygdalus pedunculata Pall* growth. A total of 827 different metabolites were detected, including 169 flavonoids, 68 organic acids, 35 terpenoids and 2 tannins. Flavonoid biosynthesis and flavone and flavonol biosynthesis were the main synthetic sources of flavonoids. Quercetin, isoquercitrin, and epicatechin as biomarkers related to growth and development were found. Quercetin connects the biosynthesis of flavonoids and the biosynthesis of flavones and flavonols. The contents of isoquercitrin and epicatechin increased uniformly during the whole growth process from the flowering stage to the fruit ripening stage, indicating that play key roles in the fruit growth and ripening stages of this plant. The tissue location and quantitative analysis of flavonoids in leaves at different stages were performed by confocal laser scanning microscopy. The flavonoids were mainly distributed in the palisade tissue and spongy tissue, indicating the need for protection of these sensitive tissues in particular. Through comprehensive and systematic analysis, the temporal distribution of flavonoids in the process of their leaves growth was determined. These results clarify the important role of flavonoids in the developmental process of *Amygdalus pedunculata* Pall.

## Introduction

Rosaceae are dicotyledonous plants, and their species resources are very abundant. It is recorded that there are more than 3,300 species in 124 genera in this family, which are widely distributed all over the world, especially in the northern temperate zone. Rosaceae plants are important landscape plants, oil plants, perfume plants and so on. There are approximately 51 genera and more than 1,000 species from this family in China (He, 2011). Among these species, The leaves, flowers, fruits and other organs of many varieties contain important bioactive substances, such as flavonoids, organic acids, and terpenoids (He, 2011), which have anticancer (Hyerin et al., 2017; Yu et al., 2017), antioxidant (Ringl et al., 2007), anti-inflammatory, bacteriostatic (Vongtau et al., 2004), and hypoglycemic (Bailie et al., 2016) properties as well as other medicinal value.

*Amygdalus pedunculata* Pall (*A. pedunculata*) is a deciduous shrub of the almond genus in the family Rosaceae and is native to arid regions in northwest China, Mongolia, and eastern Siberia. Due to its cold and drought-tolerant characteristics and deep root system, *A. pedunculata* is a candidate plant suitable for desert reclamation and can adapt to a variety of soil types and soil moisture conditions (Chu et al., 2013), which has important ecological significance for desertification control. Wild *A. pedunculata* seeds were observed to be rich in lipids, proteins, vitamins and mineral elements, and their oxygen radical absorbance capacity values indicated that the seeds were a good dietary source of antioxidants (Wang et al., 2019). The oil of *A. pedunculata* seeds lowered hyperlipidemia risk factors by improving plasma antioxidant defenses and lipid profiles (Gao et al., 2016). The polyphenols in *A. pedunculata* seed coat have antioxidant, antibacterial, and anti-HepG2 cell activities (Lu et al., 2018a). More than 300 proteins were identified in the seeds of *A. pedunculata*, and some of them have disease resistance, so *A. pedunculata* can be used as a new type of plant protein resource (Li et al., 2020).

Recently, metabolomics has been widely used in the study of plants. For example, the effects of different temperature regimes on the metabolism of three species of eucalyptus were assessed (Mokochinski et al., 2018), wheat resistance mechanisms against *Fusarium graminearum* were revealed (Su et al., 2020), the variety of *Osmanthus fragrans* flowers were classified and the antioxidant activity predicted (Zhou et al., 2017) and so on. Plants show changes in metabolic profiles with changes in their developmental stage (Zhao et al., 2015; Jeon et al., 2017). The metabolites in licorice roots were identified to illustrate regional differences, natural variations, and intra- and interspecies differences (Rizzato et al., 2017). In this study, we analyzed *A. pedunculata* leaves at different times by ultra-performance liquid chromatography quadrupole time-of-flight mass spectrometry (UPLC-QTOF-MS). Combined with chemometrics for non-targeted metabolomics analysis, the metabolic changes of several bioactive compounds during the growth *A. pedunculata* leaves were studied. In addition, confocal laser scanning microscopy (CLSM) was used to determine the tissue location and quantitative analysis of the flavonoids in leaves at different stages. Through these analyses, we clarified the changes in the important secondary metabolites of *A. pedunculata* leaves during the growth process.

## Materials and Methods

### Chemicals and Materials

Methanol, formic acid, water, and acetonitrile were all purchased from CNW Company (Düsseldorf, Germany). L-2-chlorophenylalanine was purchased from Shanghai Hengchuang Biological Technology Co., Ltd. (Shanghai, China). LysoPC17:0 was purchased from American Avanti Company (America). 2-Aminoethyl diphenylborinate was purchased from Acmec Biochemical Company (Shanghai, China). All chemicals and solvents were of analytical grade or chromatographic grade.

### Sample Collection

*A. pedunculata* was planted at the base of the Shaanxi Desert Control Research Institute in Yulin, Shaanxi, China. The leaves of *A. pedunculata* were collected manually during the flowering period, fruit formation period, fruit hard core period and fruit ripening period. The sampling time was April 18, 2019 (AP1, flowering stage), May 16, 2019 (AP2, fruit formation stage), June 2 2019 (AP3, fruit hard core stage), June 17 2019 (AP4, fruit hard core stage), July 3 2019 (AP5, fruit ripening stage), and July 19, 2019 (AP6, fruit ripening stage). Well-grown leaves were chosen for collection. The leaves collected on each sampling date were collected from the same region of different plants, and the leaves were basically the same size and shape. Six duplicate samples were collected at each time point, immediately placed into dry ice, and sent back to the laboratory for storage at −80°C.

### Sample Preparation

Eighty milligrams of sample was weighed, and 20 μL of internal standard (0.3 mg/mL L-2-chlorophenylalanine, 0.01 mg/mL Lyso PC17:0, all in methanol) and 1 mL of methanol:water (V:V = 7:3) were added. Two small steel balls were added, pre-cooled at −20°C for 2 min, added to a grinder (60 Hz, 2 min), and ultrasonically extracted for 30 min. The mixture was allowed to stand for 20 min at −20°C and centrifuged for 10 min (13,000 rpm, 4°C). A total of 300 μL of the supernatant was collected, dried and then reconstituted with 400 μL of methanol-water (V: V = 1:4). The mixture was then vortexed for 30 s, sonicated for 2 min, and centrifuged for 10 min (13,000 rpm, 4°C). A total of 150 μL of the supernatant was filtered through a 0.22 μm organic phase syringe filter, transferred to an LC injection vial, and stored at −80°C until LC-MS analysis. To optimize the LC-MS system before sample analysis and to evaluate the stability of the mass spectrometer, quality control (QC) samples were prepared from equal volumes of extracts from all samples (Lin et al., 2019). Each QC volume was the same as the sample volume. All extraction reagents were pre-cooled at −20°C before use.

### UPLC-QTOF-MS Analysis

An ACQUITY UPLC from Waters Corporation was used in series with an AB Triple TOF 5600 high-resolution mass spectrometer from AB Sciex Corporation. Chromatographic separation was performed on an ACQUITYUPLC BEH C18 column (100 mm × 2.1 mm, 1.7 μm) from Waters Corporation. The mobile phase consisted of eluent A (aqueous 0.1% formic acid solution, v/v) and eluent B (acetonitrile:methanol = 2:3 aqueous 0.1% formic acid solution, v/v). The column temperature was 45 °C, the flow rate was 0.4 mL/min, and the injection volume was 5 μL. The applied elution conditions were as follows: 0–2 min, 5% B; 2–4 min, 20% B; 4–9 min, 25% B; 9–17 min, 60% B; 17–19 min, 100% B; and 19.1–20.1 min, 5% B (King et al., 2019). The instrument was equipped with an ESI probe, and sample mass spectral signal acquisition was conducted in positive and negative ion scanning modes (Negrin et al., 2019). Mass spectrometric tuning parameters for LC-MS analysis employed optimized settings as follows: ion source temperature, 550°C; nebulizer gas and auxiliary gas flow rate, 40 PSI; curtain gas flow rate, 35 PSI; mass scan range (TOF-MS scan), m/z 70–1,000; mass scan range (product ion scan), m/z 50–1,000; and collision energy, 30 eV.

### Data Collection and Post-data Processing

UNIFI 1.8.1. software was used to collect raw data. Prior to pattern recognition, the raw data were subjected to baseline filtering, peak identification, integration, retention time correction, peak alignment, and normalization using the metabolomics processing software Progenesis QI v2.3 (Non-linear Dynamics, Newcastle, United Kingdom). An Excel file was obtained with three-dimensional data sets, including m/z values, peak retention times (RTs) and peak intensities, and RT–m/z pairs were used as the identifier for each ion. The resulting matrix was further reduced by removing any peaks with missing values (ion intensity = 0) in more than 50% of the samples. The internal standard was used for data QC (reproducibility). Metabolites were then qualitatively analyzed using HMDB (The Human Metabolome Database^1^), Lipidmaps^2^, METLIN database and self-built databases based on accurate masses, secondary fragments and isotopic distributions. After obtaining the qualitative data, the qualitatively obtained compounds were screened. Finally, the positive and negative ion data were combined.

### Statistical Analysis

For statistical analysis, the data were normalized and then introduced into the R ropls package (RStudio 1.2.1335) for pattern recognition. Multivariate statistical analysis and univariate statistical analysis were carried out to visualize the metabolic alterations among experimental groups after mean centering (Ctr) and Pareto variance (Par) scaling, respectively. Principal component analysis (PCA) was used to observe the overall distribution between samples and the stability of the entire analysis process. Orthogonal partial least squares-discriminant analysis (OPLS-DA) was then used to distinguish the overall differences in metabolic profiles between groups to find differential metabolites between groups. To prevent the model from overfitting, we used sevenfold cross validation and 200 response permutation testing (RPT) methods to examine the quality of the model. Afterward, the results used Tukey’s test (*p* < 0.05) and fold change (FC) analysis to compare the differential metabolites between the two groups.

### Confocal Laser Scanning Microscopy

CLSM is one of the most advanced techniques in microscopy and involves focusing on a specific point on a sample and obtaining images in the x/y, y/z, and x/z planes (Archana et al., 2016). According to the methods of Markham et al. (2000) and Chen et al. (2017), we evaluated the microstructure and distribution of flavonoids in the leaves of A.P. in different periods by CLSM.

The leaf tissue, which was hand-cut into thin slices (approximately 0.1 mm), was placed in 0.1 mol/L phosphate buffer (with the addition of 1% NaCl (w/v), pH = 7). An appropriately shaped section was chosen and placed onto a microscope slide with a drop of buffer solution, and an image was taken as a control. Next, the leaf tissue was stained with 0.1% 2-aminoethyl diphenylborinate (w/v) in methanol (NA solution) for 5 min, phosphate buffer (pH = 7) was used to wash off the excess staining solution, and images were taken as the experimental group. Confocal images were collected by a Nikon A1 confocal microscope used in DU4 mode with a 40£/1.2 water immersion objective. A 488 nm argon ion laser was used to excite the dye molecules. Fluorescence images were collected with emission wavelengths of 500–560 and 570–630 nm. NIS-Elements software was used to adjust and process the image acquisition parameters. Triplicate experiments were performed, and each tissue section was photographed in triplicate.

## Results

### Non-targeted Metabolomics Profiling of Different Time Samples

The samples were collected from flowering stage (AP1, April 18, 2019) to fruit ripening stage (AP6, July 19, 2019). AP2 (May 16, 2019) is the fruit formation stage. The AP3 (June 2, 2019) and AP4 (June 17, 2019) samples are both in the fruit hard core stage, and AP5 (July 3, 2019) and AP6 (July 19, 2019) samples are both in the fruit ripening stage. To explore the comprehensive changes in the *A. pedunculata* metabolome in different periods, a non-targeted metabolomics method was adopted for AP1, AP3 and AP6, and 5,187 metabolites were identified, including benzenoids, lignans, neolignans and related compounds, lipids and lipid-like molecules, nucleosides, nucleotides, and analogs, organic acids and derivatives and so on (Supplementary Table 1).

#### Principal Component Analysis

**FIGURE 1:**
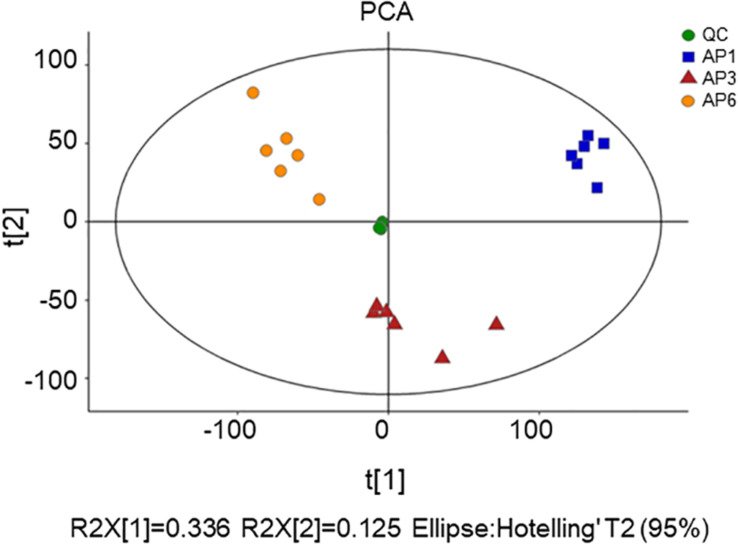
The score plots of PCA for metabolite profiles in *A. pedunculata* leaves. AP1 samples were picked at the flowering stage of the plant; AP3 samples were picked at the fruit development stage; AP6 samples were picked at the fruit ripening stage; QC was quality control sample.

**FIGURE 2:**
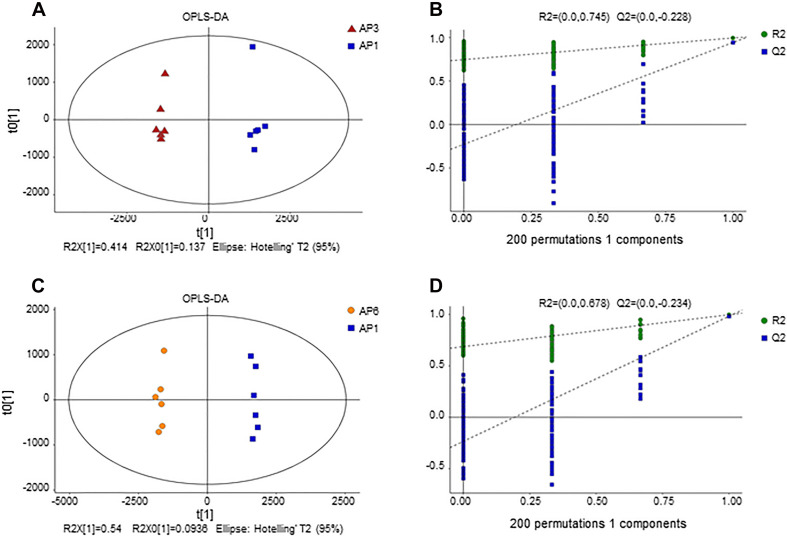
OPLS-DA score plot for samples. **(A)** OPLS-DA score plots from metabolite profiles for AP3 and AP1 samples. **(B)** A 200 times permutation test of OPLS-DA models for **(A)**. **(C)** OPLS-DA score plots from metabolite profiles for AP6 and AP1 samples. **(D)** A 200 times permutation test of OPLS-DA models for **(C)**.

First, unsupervised PCA was used to reflect the overall variability in metabolite profiles between and within the sample groups and to observe the overall distribution trend between samples (Deshpande et al., 2014). The scatter plots of PCA scores for all samples are shown in Figure 1. Four principal components of the PCA plot explained 55.3% of the total data variance. As seen from Figure 2, all samples were within the 95% confidence interval (Ellipse: Hotelling ’T2). The QC samples were tightly clustered, indicating that the experiment was stable and repeatable. From PCA, it can be seen intuitively that there were obvious differences in the leaf metabolites at three time points.

#### Orthogonal Partial Least Squares-Discriminant Analysis

In order to establish a more predictive model, we performed supervised OPLS-DA on the basis of PCA to enhance the difference between the experimental group and the control group. The scatter plot of OPLS-DA scores for all samples is shown in Figure 2. The AP3 and AP6 samples showed obvious metabolic differences compared with the AP1 samples (Figures 2A,C). The OPLS-DA models were well constructed with high R2Y and Q2 values (Table 1), which indicated an excellent fit and satisfactory predictive power. In this study, a 200-time permutation test was performed. The R2-intercepts for the AP3 and AP6 samples were 0.745 and 0.687, respectively, whereas the Q2-intercepts were −0.228 and −0.234, respectively (Figures 2B,D), indicating that the PLS-DA model showed no overfitting and was credible.

**TABLE 1:** Each parameter of PCA model.

Group	Type	PRE	ORT	N	R2X (cum)	R2Y (cum)	Q2 (cum)	R2	Q2
AP3/AP1	PCA	2	0	12	0.553	–	–	–	–
AP3/AP1	OPLS	1	1	12	0.550	0.993	0.941	0.745	−0.228
AP6/AP1	PCA	2	0	12	0.636	–	–	–	–
AP6/AP1	OPLS	1	1	12	0.634	0.997	0.982	0.687	−0.234

### Variations in Differential Metabolites of Samples in Different Periods

According to the OPLS-DA model, variable importance of projection (VIP) values were obtained. Differential metabolites were selected on the basis of the combination of a statistically significant threshold of VIP values obtained from the OPLS-DA model and *p*-values from a two-tailed Student’s *t*-test on the normalized peak areas, where metabolites with VIP values greater than 1.0 and *p*-values less than 0.05 were considered differential metabolites (Wang et al., 2018). A total of 827 differential metabolites were obtained after screening. Among these metabolites, a total of 274 bioactive metabolites, there are 169 flavonoids, 68 organic acids, 35 terpenoids, and 2 tannins (total 274 metabolites) were obtained (Supplementary Table 1). Comparison of samples AP1 and AP3 obtained 641 differential metabolites, with 334 upregulated and 307 downregulated. Comparison of samples AP1 and AP6 obtained 675 differential metabolites, with 280 upregulated and 395 downregulated metabolites.

### Comparison of Different Metabolite Pathway Analysis of Samples in Different Periods

**FIGURE 3:**
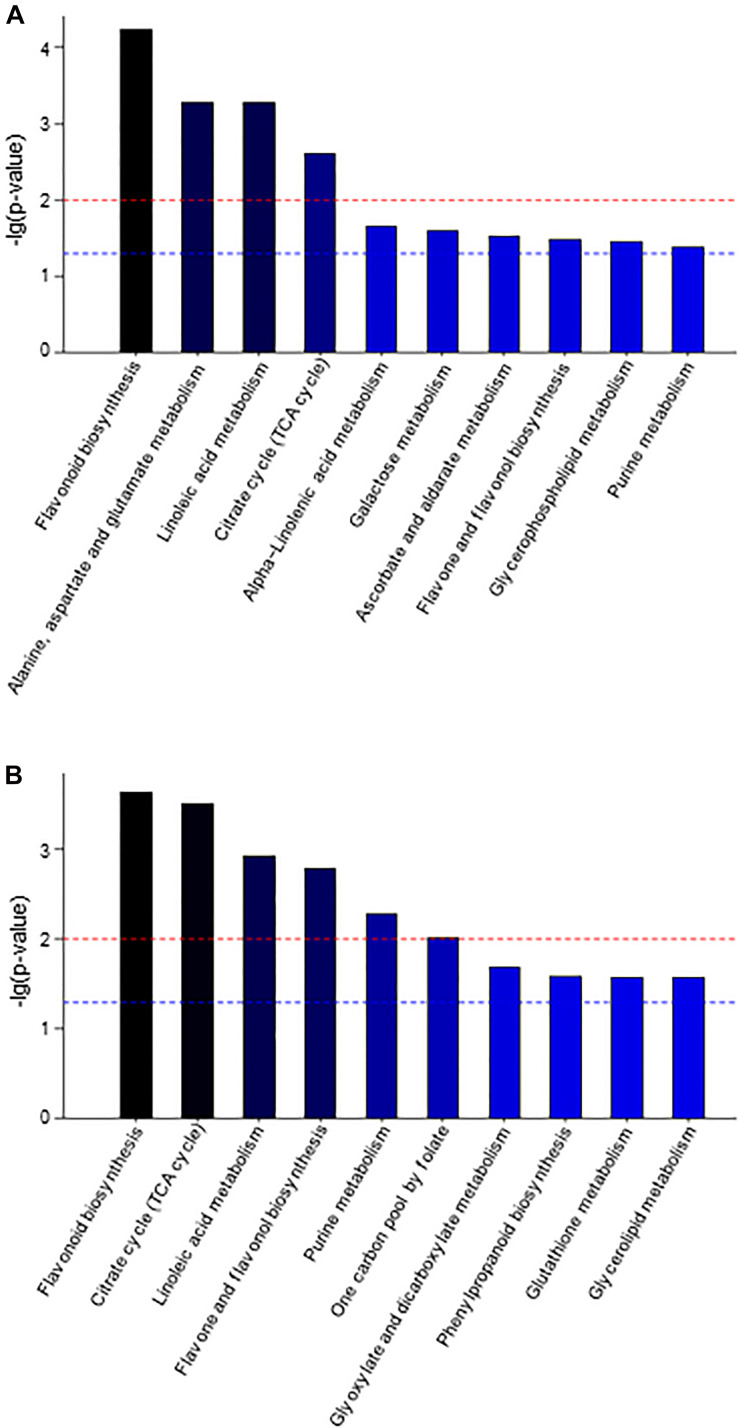
The KEGG pathway enrichment analysis of differential metabolites between sample AP3 versus sample AP1 **(A)** and sample AP6 versus sample AP1 **(B)**. Each comparison mycelia only shows top 10 enrichments pathways of differential metabolites. *P*-value was calculated using hypergeometric test.

To understand the differences in the metabolic network between the three groups of samples, the differential metabolites were submitted to the KEGG (Kyoto Encyclopedia of Genes and Genomes^3^) website for metabolic pathway enrichment analysis. Figure 3 shows the top ten enrichment pathways for the −lg (*p*-value) values for samples AP3 and AP6 in comparison with sample AP1, including three common primary metabolic pathways [citrate cycle (TCA cycle), purine metabolism and linoleic acid metabolism] and two common specialized metabolic pathways (flavonoid biosynthesis and flavone and flavonol biosynthesis). In the AP3/AP1 group, the metabolic pathways of flavonoid biosynthesis ranked first, and flavone and flavonol biosynthesis ranked eighth. In the AP6/AP1 group, the metabolic pathways of flavonoid biosynthesis still ranked first, and flavone and flavonol biosynthesis ranked fourth. These results showed that the pathways flavonoid biosynthesis and flavone and flavonol biosynthesis were significantly different from the flowering stage to the fruiting stage of the plant.

### Accumulation Dynamics of Flavonoids by CLSM

**FIGURE 4:**
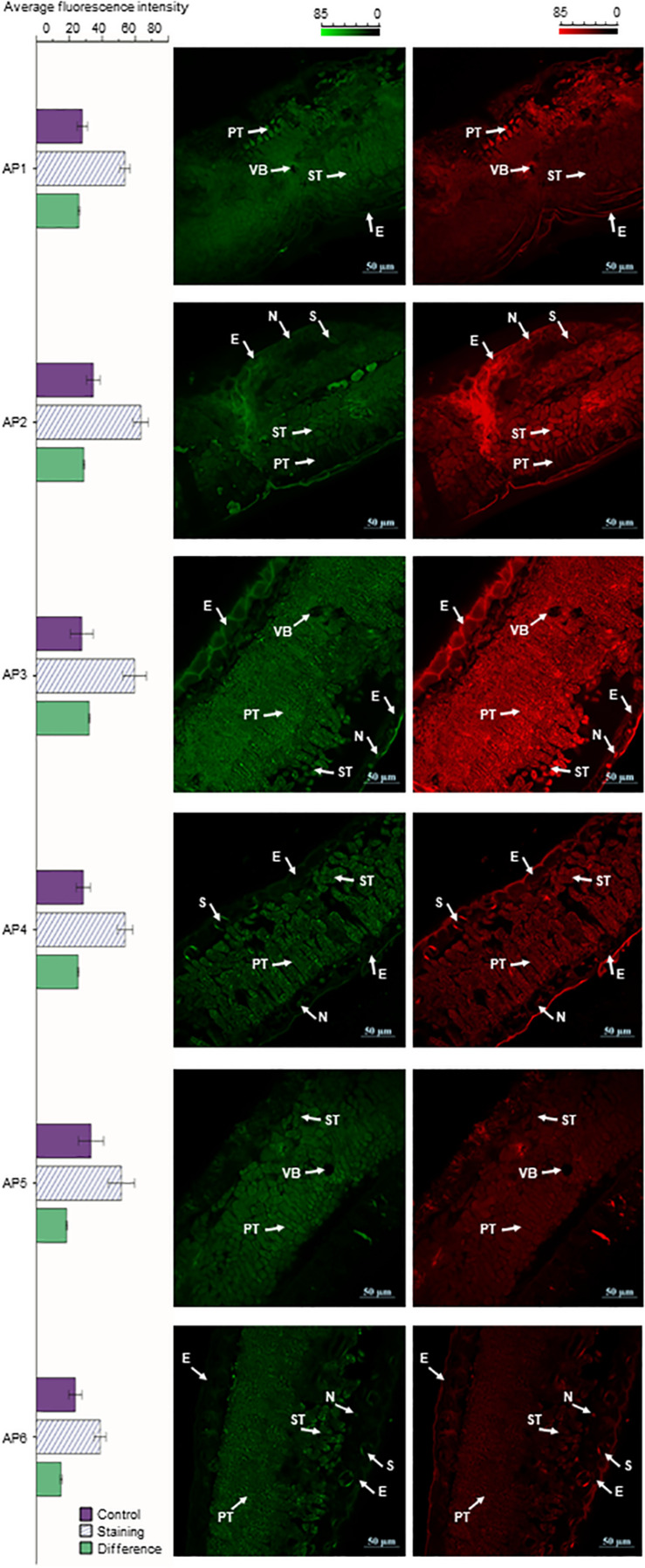
*A. pedunculata* leaves of different periods were stained with NA solution and observed with CLSM (bar = 50 μm). The distribution of flavonoids in the cross-section of the *A. pedunculata* leaves after dyeing is shown. Three independent experiments were performed, and each experiment was tested three times in parallel. Calculate the average fluorescence intensity, and the result *p* < 0.01 (E, epidermis; N, nucleolus; VB, vascular bundle; PT, Palisade tissue; ST, Spongy tissue; S, stomata).

The cross-sections of *A. pedunculata* leaves stained with NA (Naturstoffreagenz A) solution in different periods were observed by CLSM. At an excitation wavelength of 488 nm, flavonols and flavonoid glycosides emitted green and red fluorescence. The samples showed chloroplast autofluorescence before staining, and this interference was eliminated by subtracting the background signal. The laser confocal image of the leaf cross section shows that the fluorescence intensity was higher in the spongy tissue and palisade tissue than in other tissue of the leaves. Moreover, the fluorescence intensity of guard cells on both sides of the stomata and the nucleolar area of epidermal cells were relatively high. However, the fluorescence intensity in the cytoplasm of epidermal cells was relatively low, and the fluorescence intensity in the vascular bundle was lowest. The relative fluorescence intensity reached a peak in AP3 samples, and the relative fluorescence intensity was the smallest in the AP6 samples. The relative fluorescence intensity of the AP3 sample was 2.13 times that of AP6, the relative fluorescence intensity of the AP3 sample was 1.24 times that of AP1, and the relative fluorescence intensity of the AP6 sample was 0.58 times that of AP1 (Figure 4).

## Discussion

As a member of the Rosaceae family, *A. pedunculata* is a sandy and stable oily wood. This species possesses important economic and ecological protection value. More interestingly, some organs of *A. pedunculata* also have significant nutrients and active substances. *A. pedunculata* seeds contain high levels of phenols, tocopherols, fiber and phytosterols, as well as compounds with antioxidant activity, including vitamin E and sphingolipids, which have recently attracted significant attention from the nutraceutical industry (Chen et al., 2006; Sorkheh et al., 2016). The essential oil from *A. pedunculata* leaves extracted by the ultrasonic-assisted method can scavenge 2,2-diphenyl-1-picrylhydrazyl (DPPH) radicals, hydroxyl (OH⋅) radicals, hydrogen peroxide (H_2_O_2_) and superoxide anion radicals (O_2_^–^) (Lu et al., 2018b). However, many active substances vary greatly in different stages of plant growth and development. Therefore, it is important to determine the content of active substances at different growth stages. However, the characteristics of bioactive compound changes in *A. pedunculata* leaves at different times have not been studied. In this study, we used UPLC-MS to detect metabolites at different times and applied the metabolomics approach to statistically analyze the metabolic datasets to obtain changing situations in several bioactive compounds.

### Metabolic Analysis of *A. pedunculata* Leaves

Since secondary metabolites often play a very important role in the growth and development of plants, and they change significantly in different growth stages of plants, we focused on the changes of secondary metabolites during plant growth and development. A total of 827 differential metabolites were obtained from 5187 metabolites. Among these metabolites, a total of 274 specialized metabolites, including 169 flavonoids, 68 organic acids, 35 terpenoids and 2 tannins, were shown and discussed in Supplementary Table 2.

Flavonoids are widely present in the leaves, fruits, roots, and skin of plants. These compounds play a variety of physiological roles in plant growth, development, and reproduction (Deng et al., 2018), and they are good plant growth regulators and plant antitoxins (Yoshioka et al., 2004). Flavonoids also play significant roles in plant defense against phytopathogens and in auxin transport regulation (Kumar and Pandey, 2013; Petrussa et al., 2013; Deng and Lu, 2017). A large number of studies have shown that flavonoids have many pharmacological effects, such as antibacterial, antirheumatic, antiallergic, antitumor, antiviral, anticonvulsant, analgesic, antioxidant, and antiaging effects (Ahangari et al., 2018). Flavonoids also have the properties of protecting the liver and nervous system and preventing cardiovascular and cerebrovascular diseases (Zhang et al., 2016).

This study found that the leaves of *A. pedunculata* are rich in flavonoids, including flavonoid glycosides, flavones, biflavonoids and polyflavonoids, homoisoflavans, and isoflavans (Supplementary Table 2). AP3/AP1 contained 126 metabolites (44 downregulated metabolites and 82 upregulated metabolites), and AP6/AP1 contained 126 metabolites (71 downregulated metabolites and 55 upregulated metabolites). Flavonoids are mostly related to the plant’s own defense system (Vicente and Boscaiu, 2018). Due to the AP3 sample being collected during the fruit growth and development stage, the content of flavonoids in *A. pedunculata* in the AP3 stage was very high, it was speculated that the increase in the flavonoids content may have the effect to prevent pests and bacterial infection during the fruit hard core stage.

Organic acids are widely distributed in the leaves, roots, and fruits of plants (Mikulic-Petkovsek et al., 2012). Some natural organic acids, such as citric acid, malic acid, tartaric acid, ascorbic acid, etc., have bacteriostatic, anti-inflammatory, hypoglycemic, antioxidant, and immune-modulating effects (Pande and Akoh, 2010). Some special organic acids are effective ingredients of certain Chinese herbal medicine (Ahangari et al., 2018). In this study, many significant organic acids were detected in *A. pedunculata* leaves (Supplementary Table 2). AP3/AP1 contained 54 metabolites (41 downregulated metabolites and 13 metabolites upregulated), and AP6/AP1 contained 57 metabolites (42 downregulated metabolites and 15 upregulated metabolites). Terpenoids can improve the disease resistance and antifungal ability of plants. These compounds are important secondary metabolites in plants and play an important role in the normal physiological activities of plants (Chen et al., 2018). Twenty-three significantly different terpenoids (5 downregulated and 18 upregulated) were obtained in AP3/AP1 samples, and 25 significantly different terpenoids (5 downregulated and 20 upregulated) were obtained in the AP6/AP1 samples. Data are shown in Supplementary Table 2, including four types of substances: monoterpenoids, terpene lactones, triterpenoids, and terpene glycosides. Tannins are a class of polyphenolic compounds with complex structures. Tannins are widely distributed in plants, especially in bark (Garcia et al., 2013), and they are the basis of the bioactive substances of many commonly used medicines (Ajebli and Eddouks, 2019). A total of two tannin compounds were obtained from the different metabolites of A.P. leaves in different periods, namely, 3,4-hexahydroxydiphenoylarabinose (AP6/AP1) and beta-glucogallin (AP3/AP1) (Supplementary Table 2).

### Metabolic Pathways of Flavonoids

**FIGURE 5:**
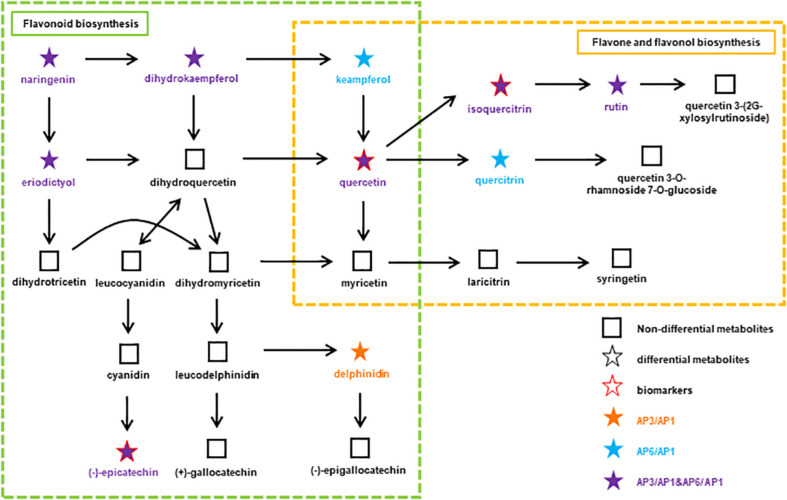
At different growth stages, the flavonoids metabolic pathway in *A. pedunculata* leaves. The orange dot indicates that the substance is present in the AP3/AP1 samples; the blue dots indicate that the substances are present in the AP6/AP1 samples; the purple dots indicate that the substances are present in both samples.

Among the several types of bioactive differential metabolites studied, flavonoids were the most abundant in *A. pedunculata* leaves. Flavonoid metabolism in leaves is related to the growth period. Compared with those in the AP6 samples, flavonoids were more abundant in the AP3 samples. Therefore, we speculated that during the fruit development period, the plant’s defense ability against foreign invasion was higher than that during the other two periods. At different growth stages, the changes in flavonoid metabolite markers in leaves appeared as a metabolic pathway. After correlation analysis of key metabolites and growth stages, some differential metabolites with important significance were selected and applied for metabolic network pathway analysis. Figure 5 shows a flavonoid pathway map reporting key points at which metabolism changed in the metabolic pathways using the connectivity matrix (see text footnote 3).

There are two metabolic pathways related to flavonoids: flavonoid biosynthesis and flavone and flavonol biosynthesis. Among them, the metabolic pathway of flavonoid biosynthesis was highly significant in the AP3/AP1 and AP6/AP1 groups (*p* < 0.01). The metabolic pathway of flavonoid and flavonol biosynthesis was only significant in the AP6/AP1 group. There were 11 metabolites related to these two pathways: naringenin, delphinidin, epicatechin, holy phenol, pinusin, quercetin, isoquercitrin, rutin, mountain kaempferol, asperin, and quercetin. These substances have an important influence on the synthesis of flavonoids, and they are synthesized by plants to serve many actions as visual attractors, feeding repellents, photoreceptors, oviposition stimulants, allelopathy, phytoalexins, antioxidants, and antimicrobials (Jeandet et al., 2013, 2014). Among them, quercetin, isoquercitrin and epicatechin are considered as potential biomarkers closely related to the growth and development of *A. pedunculata*. Due to isoquercitrin and epicatechin were upregulated throughout the plant growth process, indicating that they play a key role in plant growth and fruiting. Studies have shown that the ratios of the “effective antioxidants” quercetin and luteolin glycosides to the “poor antioxidants” kaempferol and apigenin glycosides significantly increase upon high light irradiance, irrespective of the relative proportions of different solar wavelengths reaching the leaf surface (Agati and Tattini, 2010; Pollastri and Tattini, 2011). Quercetin 3-O- and luteolin 7-O-glycosides accumulate similarly in response to UV-B irradiance or root zone salinity in *Ligustrum vulgare* (Agati et al., 2011). From the AP1 period to the AP3 period, the local climate caused the light intensity to gradually increase. Except for naringenin and eriodictyol, the content of the other 9 compounds increased significantly, indicating that these substances play an important role in protecting plants from strong UV-B radiation. In particular, quercetin is very important for the synthesis of flavonoids. It connects the two pathways of flavonoid biosynthesis and flavone and flavonol biosynthesis. Quercetin and its derivatives can protect chloroplasts from the production of singlet oxygen (1O2) induced by visible light (Agati et al., 2007).

### Distribution of Flavonoids in Mesophyll Cells Revealed by CLSM

Through LSCM analysis, we can more clearly see the difference in temporal and spatial distribution of flavonoids in *A. pedunculata* leaves. The leaf section was clearly divided into three areas, and the content of flavonoids in different types of cells was obviously different. The location of flavonoids within different cells and cellular compartments is potentially related to their multiple functions in plant environment interactions (Agati et al., 2012). Since flavonoids are synthesized via a well-characterized multienzyme complex localized in the cytoplasmic surface of the endoplasmic reticulum, efficient flavonoid transport systems deliver these metabolites across different membrane-limited compartments (Zhu et al., 2016). Because some phenylpropanoids and flavonoids are secreted outside of the cell, the nuclear and cell wall fluorescence intensities of the epidermal cells in the leaves were relatively high, so the content of flavonoids in the epidermal nucleus was relatively high, but there was almost no fluorescence in the cytoplasm. The increase in cell wall flavonoids as leaves aged was paralleled by a decrease in soluble flavonoids. This suggested that vacuolar efflux of these metabolites and deposition in the cell wall occurred (Zhao and Dixon, 2010). Quercetin and kaempferol derivatives have also been observed in the cell wall of epidermal cells in lisianthus flower petals (Markham et al., 2000). Moreover, phenylpropanoids, particularly hydroxycinnamic acid derivatives, contribute to cell wall formation through esterification with complex carbohydrates.

Chloroplasts have long been reported to contain flavonoids and appear to be capable of flavonoid biosynthesis (Hernandez et al., 2009; Pollastri and Tattini, 2011). In the *A. pedunculata* leaves, we discovered the main accumulation of flavonoids in the palisade tissue and spongy tissue, indicating the need for protection of these sensitive tissues (Sheahan, 1996; Karabourniotis et al., 1998). Since flavonoids can be transported over long distances by vascular bundles, the content of flavonoids in leaves increased during the hard core stage (AP3), and the temperature and light duration increases during this period. Plants transport flavonoids to fruit, protecting fruit from ultraviolet radiation damage. At the fruit ripening stage (AP6), the flavonoid content was reduced. Through microstructural analysis, we also found that *A. pedunculata* leaves contained developed stratum corneum and vascular bundles, tightly arranged palisade tissue, and a large number of stomata. In addition, previous reports have found that in hostile environments, a dense covering of wax in several species might help to reduce water loss by lowering the amount of light absorbed by the leaf and accompanying heat load (Feucht et al., 2005). Combined with the conclusions described above, the acclimation ability of *A. pedunculata* in hostile environments (such as intensive irradiation, deficiency of water) can be well proven.

## Conclusion

This study provides a comprehensive evaluation of the temporal changes in bioactive metabolites in *A. pedunculata* at different growth stages, especially the types and biosynthetic pathways of flavonoids. CLSM was used to analyze the tissue location of the flavonoids contained in *A. pedunculata* leaves at different times. It was quantitatively verified by fluorescence intensity that the fruit development stage had the highest flavonoid content in *A. pedunculata* leaves. We clarified the key role of flavonoids in the different growth and developmental stages of *A. pedunculata* leaves. This study provides a theoretical basis for future research on artificially increasing the content of flavonoids in *A. pedunculata* leaves. In addition, this study will provide a theoretical basis and scientific basis for the development of the social benefits of *A. pedunculata* as a medicinal material and the economic effects of edible medicinal plants, increasing comprehensive utilization value of *A. pedunculata*.

## Data Availability Statement

The original contributions presented in the study are included in the article/Supplementary Material, further inquiries can be directed to the corresponding author/s.

## Author Contributions

YS, CL, and YH conceived and designed the research. YH and LP searched for relevant literature. YH, LP, and TY performed the experiments. CL and YH analyzed the experimental data and wrote the manuscript. BC, WW, and LP assisted in analyzing the experimental data. YS and CL guided the manuscript preparation. All authors read and approved the manuscript.

## Conflict of Interest

The authors declare that the research was conducted in the absence of any commercial or financial relationships that could be construed as a potential conflict of interest.

## Footnotes

1 http://www.hmdb.ca/

2 http://www.lipidmaps.org/

3 http://www.genome.jp/kegg/pathway.html
