# Supplementary material for: Metabolomic and Confocal Laser Scanning Microscopy (CLSM) Analyses Reveal the Important Function of Flavonoids in Amygdalus pedunculata Pall Leaves With Temporal Changes
Source: Front Plant Sci. 2021 May 19;12:648277. doi: 10.3389/fpls.2021.648277 (PMC8170035; doi:10.3389/fpls.2021.648277)
Supplement: Supplementary file 2 [file Table_2.docx]

Table 2: Identification of 274 bioactive different metabolites by UPLC–QTOF–MS from A.P. leaves

|  | **Metabolites** | **m/z** | **VIP(AP**  **3/AP1)** | **VIP(AP**  **6/AP1)** | **FC(AP**  **3/AP1)** | **FC(AP**  **6/AP1)** |
| --- | --- | --- | --- | --- | --- | --- |
| **Flavonoids (169)** | | | | | | |
| 1 | Pinocembrin | 257.08 | 1.07 | - | 2.70 | - |
| 2 | (-)-Naringenin | 273.08 | 2.56 | 2.06 | 0.09 | 0.12 |
| 3 | Kaempferol | 285.04 | - | 2.72 | - | 0.32 |
| 4 | Aromadendrin | 287.06 | 3.97 | 3.81 | 0.31 | 0.15 |
| 5 | Eriodictyol | 287.06 | 2.03 | 1.45 | 0.03 | 0.16 |
| 6 | 3,5,6,7-tetrahydroxy-2-phenyl-3,4-dihydro-2H-1-benzopyran-4-one | 287.06 | 1.88 | 1.73 | 0.13 | 0.05 |
| 7 | 6,12-Methano-6H,12H-dibenzo[b,f][1,5]dioxocin-2,3,9,13-tetrol | 289.07 | - | 1.23 | - | 0.46 |
| 8 | Epicatechin | 291.09 | 5.10 | 3.05 | 2.67 | 1.90 |
| 9 | Quercetin | 303.05 | 2.05 | 2.51 | 1.24 | 0.62 |
| 10 | 3,5,6,7-tetrahydroxy-2-(3,4,5-trihydroxyphenyl)-3,4-dihydro-2H-1-benzopyran-4-one | 319.04 | 1.04 | - | 129.89 | - |
| 11 | 3-(2,3-dihydroxy-4-methoxyphenyl)-7-hydroxy-3,4-dihydro-2H-1-benzopyran-4-one | 320.13 | 1.03 | - | 3.91 | - |
| 12 | 2'-Hydroxy-2,3,4',6'-tetramethoxychalcone | 345.13 | 1.05 | 1.06 | 2.95 | 3.57 |
| 13 | 5-Methyl-6-hydroxyluteolinidin | 348.08 | 2.11 | 2.23 | 0.33 | 0.10 |
| 14 | Moschamindole | 349.12 | - | 1.94 | - | 161.11 |
| 15 | Laurifolin (flavonoid) | 357.13 | - | 1.30 | - | 9.33 |
| 16 | 5,2',3'-Trihydroxy-3,7,8-trimethoxyflavone | 361.09 | 1.97 | 1.73 | 0.42 | 0.38 |
| 17 | Lilaline | 401.13 | 2.00 | 1.97 | 5.44 | 6.44 |
| 18 | Distemonanthin | 403.03 | 1.17 | 1.22 | 2.75 | 3.77 |
| 19 | Catechin 7-O-beta-D-xyloside | 403.10 | 1.02 | - | 0.16 | - |
| 20 | Vestitone 7-glucoside | 417.15 | 2.74 | 3.13 | 9.27 | 14.39 |
| 21 | 4'-Methylliquiritigenin 7-rhamnoside | 417.15 | 2.53 | 3.59 | 29.70 | 73.95 |
| 22 | Afzelechin 4'-O-beta-D-glucopyranosdie | 419.13 | 2.03 | - | 2.83 | - |
| 23 | Avicularin | 433.08 | - | 6.30 | - | 0.71 |
| 24 | Dihydroamorphigenin | 435.14 | 1.04 | - | 0.31 | - |
| 25 | Loquatoside | 439.12 | 1.23 | - | 6.99 | - |
| 26 | 6-Hydroxykaempferol 3-glucoside | 445.08 | 1.12 | - | 9.62 | - |
| 27 | Dichotosin | 447.16 | 1.23 | 1.09 | 0.10 | 0.03 |
| 28 | Quercitrin | 449.11 | - | 2.66 | - | 0.63 |
| 29 | Pectolinarigenin 7-glucoside | 459.13 | 2.03 | - | 3.28 | - |
| 30 | Isoquercitrin | 465.10 | 7.17 | 4.32 | 2.43 | 1.81 |
| 31 | Quercetin 3-O-glucoside | 465.10 | 3.26 | 1.87 | 1.44 | 0.75 |
| 32 | Quercetin 3-galactoside | 465.10 | 1.99 | - | 3.72 | - |
| 33 | Echioidinin 2'-(6''-acetylglucoside) | 471.13 | - | 1.03 | - | 376.29 |
| 34 | Quercetin 3-(3''-acetyl-alpha-L-arabinofuranoside) | 475.09 | 1.38 | - | 2.20 | - |
| 35 | Quercetin 3,3'-dimethyl ether 7-glucoside | 475.12 | 2.37 | 2.64 | 0.41 | 0.19 |
| 36 | 3''-O-Acetylafzelin | 475.12 | 6.37 | - | 21.06 | - |
| 37 | Vitexin 2''-acetate | 475.12 | - | 1.52 | - | 0.34 |
| 38 | 6''-O-Acetylglycitin | 487.12 | 1.85 | - | 6.32 | - |
| 39 | Quercetin 3-(3''-acetylrhamnoside) | 489.10 | 8.41 | 2.69 | 21.06 | 3.55 |
| 40 | Kaempferol 3-(6-acetylgalactoside) | 489.10 | - | 2.76 | - | 0.21 |
| 41 | Isorhamnetin 3-(6''-acetylglucoside) | 519.11 | - | 1.52 | - | 0.12 |
| 42 | Artonin O | 520.23 | 2.21 | 1.96 | 0.16 | 0.11 |
| 43 | Ikarisoside A | 523.16 | 1.04 | - | 0.06 | - |
| 44 | (-)-Epigallocatechin 3'-glucuronide | 527.10 | 1.83 | 1.64 | 5.29 | 5.03 |
| 45 | Parthenosin | 533.13 | 1.84 | - | 4.15 | - |
| 46 | Cucumerin B | 533.15 | 1.51 | - | 2.53 | - |
| 47 | 6''-Malonylastragalin | 535.11 | - | 2.91 | - | 0.19 |
| 48 | Leucadenone A | 539.21 | 2.41 | 2.65 | 1.82 | 2.36 |
| 49 | Delphinidin 3-(acetylglucoside) | 552.10 | 2.17 | - | 20.15 | - |
| 50 | 2'',4'',6''-Triacetylglycitin | 553.14 | - | 1.01 | - | 271.56 |
| 51 | Biochanin A 7-O-apiosyl-(1->6)-glucoside | 561.16 | 2.40 | - | 3.21 | - |
| 52 | 8-C-Glucopyranosylgenistein 6''-O-apioside | 565.15 | - | 17.25 | - | 0.41 |
| 53 | Carpelastofuran | 567.23 | 1.08 | 1.02 | 0.40 | 0.34 |
| 54 | Triuvaretin | 571.22 | 1.00 | 1.23 | 2.08 | 2.87 |
| 55 | Eriodictyol 7-(6-trans-p-coumaroylglucoside) | 577.13 | 8.90 | 6.54 | 2.26 | 1.98 |
| 56 | Proanthocyanidin A2 | 577.13 | 3.52 | 2.20 | 4.56 | 3.21 |
| 57 | Epicatechin-(4beta->8)-gallocatechin | 577.13 | 2.98 | 1.71 | 4.16 | 2.64 |
| 58 | Quercetin 3-[rhamnosyl-(1->2)-alpha-L-arabinopyranoside] | 579.13 | 3.78 | 4.85 | 0.65 | 0.37 |
| 59 | Procyanidin B2 | 579.15 | 6.44 | - | 3.25 | - |
| 60 | Procyanidin | 579.15 | 9.61 | 5.79 | 2.82 | 2.03 |
| 61 | Viscutin 2 | 591.15 | 2.52 | 2.27 | 0.39 | 0.35 |
| 62 | Piperitoside | 593.13 | 1.38 | 1.24 | 0.56 | 0.50 |
| 63 | Kuwanon Z | 593.15 | - | 1.68 | - | 0.50 |
| 64 | Kaempferol 3-neohesperidoside | 593.15 | 9.16 | 10.20 | 0.63 | 0.45 |
| 65 | 6-Hydroxyluteolin 7-sambubioside | 595.13 | 1.76 | 2.03 | 0.56 | 0.27 |
| 66 | 5,3',4'-Trihydroxy-7-methoxy-4-phenylcoumarin 5-O-xylosyl-(1->6)-glucoside | 595.16 | 10.36 | 17.78 | 0.80 | 0.38 |
| 67 | Kaempferol 3-[2'''-acetyl-alpha-L-arabinopyranosyl-(1->6)-galactoside] | 603.13 | 1.21 | - | 10.42 | - |
| 68 | Isorhamnetin 3-O-[b-L-rhamnofuranosyl-(1->6)-D-glucopyranoside] | 605.15 | - | 1.72 | - | 0.16 |
| 69 | Isoscutellarein 4'-methyl ether 7-allosyl-(1->2)-glucoside | 605.15 | 1.55 | 1.47 | 0.18 | 0.05 |
| 70 | Isoscoparin 2''-O-glucoside | 607.16 | 2.17 | 2.25 | 0.31 | 0.11 |
| 71 | Kaempferol 5-methyl ether 3-galactoside-4'-glucoside | 607.16 | 1.94 | 2.55 | 0.52 | 0.16 |
| 72 | Chrysin 7-[rhamnosyl-(1->4)-glucoside] | 607.16 | 1.47 | - | 3.70 | - |
| 73 | Isorhamnetin 3-rhamnoside-7-glucoside | 607.16 | - | 4.47 | - | 0.30 |
| 74 | Isorhamnetin 3-O-[b-D-glucopyranosyl-(1->2)-a-L-rhamnopyranoside] | 607.16 | - | 1.59 | - | 0.27 |
| 75 | Multinoside A | 609.14 | - | 11.41 | - | 0.65 |
| 76 | Rutin | 611.16 | 5.31 | 7.76 | 1.18 | 0.60 |
| 77 | Phloretin xylosyl-galactoside | 613.18 | 1.17 | - | 5.42 | - |
| 78 | Mulberrofuran E | 613.23 | 1.81 | 2.09 | 8.76 | 11.99 |
| 79 | Biorobin | 617.15 | 1.32 | 1.32 | 0.27 | 0.06 |
| 80 | Sutchuenoside A | 619.16 | - | 10.26 | - | 0.50 |
| 81 | Vitexin 2''-O-rhamnoside-4'''-acetate | 621.18 | 17.48 | - | 2.19 | - |
| 82 | 5,4'-Dihydroxy-6-C-prenylflavanone 4'-xylosyl-(1->2)-rhamnoside | 625.23 | 1.03 | - | 11.13 | - |
| 83 | Myricetin 3-neohesperidoside | 627.15 | 1.64 | 1.17 | 3.68 | 3.36 |
| 84 | Hypoletin 8-gentiobioside | 627.16 | 1.83 | - | 2.66 | - |
| 85 | Scutellarein 7-glucuronosyl-(1->2)-glucuronide | 637.10 | 1.64 | 2.09 | 0.64 | 0.37 |
| 86 | Anhydroicaritin 3-rhamnosyl-(1->2)-rhamnoside | 643.24 | 1.10 | - | 0.05 | - |
| 87 | Scutellarein 6,7-dimethyl ether 4'-rutinoside | 645.18 | 1.22 | - | 3.92 | - |
| 88 | Dicliripariside C | 645.18 | 1.05 | - | 96.12 | - |
| 89 | Tricetin 3'-methyl ether 7,5'-diglucuronide | 649.10 | 1.02 | - | 2.50 | - |
| 90 | 8-Hydroxyluteolin 7-[6'''-acetylallosyl-(1->2)-glucoside] | 649.14 | 2.38 | - | 5.96 | - |
| 91 | Dillenetin 5-glucoside-7-glucuronide | 649.14 | 1.45 | 1.20 | 0.08 | 0.11 |
| 92 | Pinocembrin 7-O-neohesperidoside 6''-O-acetate | 651.19 | 8.66 | 8.82 | 2.09 | 2.57 |
| 93 | Kaempferol 3-rhamnoside 7-galacturonide | 653.13 | 1.94 | 1.09 | 4.89 | 3.24 |
| 94 | 2''-O-Acetylrutin | 653.17 | 9.27 | - | 1.48 | - |
| 95 | Delphinidin 3-rutinoside | 656.16 | 1.18 | 1.18 | 0.25 | 0.07 |
| 96 | Neohesperidin dihydrochalcone | 657.20 | - | 3.22 | - | 9.49 |
| 97 | Sagittatoside A | 659.23 | - | 1.27 | - | 362.46 |
| 98 | Pelargonidin 3-(6''-acetylglucoside)-5-glucoside | 660.17 | 3.70 | 3.16 | 0.01 | 0.00 |
| 99 | Desmanthin 1 | 661.11 | 1.04 | 1.63 | 0.64 | 0.25 |
| 100 | Quercetin 3-(2-galloylglucoside) | 661.11 | 1.78 | 2.01 | 0.47 | 0.20 |
| 101 | Wanepimedoside A | 661.25 | 1.69 | 1.43 | 0.00 | 0.00 |
| 102 | Scutellarein 6,4'-dimethyl ether 7-(3'''-acetylrutinoside) | 663.19 | - | 1.01 | - | 0.30 |
| 103 | 5,7-Dihydroxy-8-3',4',5'-tetramethoxyflavone 5-O-rhamnoside-6-C-glucoside | 663.19 | 1.89 | - | 1.69 | - |
| 104 | Apigenin 7-(3''-acetyl-6''-E-p-coumaroylglucoside) | 665.15 | 1.18 | - | 0.43 | - |
| 105 | 5,2',4'-Trihydroxy-3,7,5'-trimethoxyflavone 2'-galactosyl-(1->4)-glucoside | 665.17 | 1.42 | 1.30 | 0.59 | 0.56 |
| 106 | Apigenin 7-glucuronosyl-(1->2)-glucuronide | 667.11 | 1.98 | 1.31 | 5.94 | 4.19 |
| 107 | Euphorbianin | 669.17 | 4.29 | 2.75 | 59.00 | 37.56 |
| 108 | Mearnsetin 3,7-dirhamnoside | 669.17 | 1.10 | 1.16 | 2.69 | 3.31 |
| 109 | Myricetin 7-(6''-galloylglucoside) | 677.10 | - | 1.61 | - | 0.24 |
| 110 | 8-C-Ascorbylepigallocatechin 3-gallate | 677.10 | 1.03 | 1.06 | 0.38 | 0.18 |
| 111 | Okanin 4'-(4''-acetyl-6''-p-coumarylglucoside) | 683.16 | 1.70 | - | 2.68 | - |
| 112 | Apigenin 6-C-glucosyl-7-O-(6-malyl-glucoside) | 691.15 | 2.45 | 2.31 | 2.44 | 3.22 |
| 113 | Nevadensin 5-gentiobioside | 691.19 | 1.12 | - | 6.16 | - |
| 114 | Muscomin | 691.21 | - | 1.15 | - | 70.19 |
| 115 | Kuwanon K | 691.22 | 1.05 | 1.55 | 1.88 | 2.96 |
| 116 | Primflasine | 693.16 | - | 1.31 | - | 2.06 |
| 117 | Hesperetin 3',7-O-diglucuronide | 699.14 | 4.05 | 2.88 | 7.45 | 5.66 |
| 118 | 2'',3''-Di-O-p-coumaroylafzelin | 705.16 | 2.75 | 3.13 | 0.57 | 0.33 |
| 119 | 8-Hydroxyluteolin 4'-methyl ether 7-(6'''-acetylallosyl)(1->2)(6''-acetylglucoside) | 705.16 | 3.78 | 4.45 | 0.52 | 0.27 |
| 120 | Isorhamnetin 3-(4''',6'''-diacetylglucosyl) (1->3)-galactoside | 705.17 | 1.49 | 1.59 | 0.44 | 0.29 |
| 121 | Apigenin 7-(6''-malonylneohesperidoside) | 709.16 | 4.38 | 2.21 | 7.72 | 4.05 |
| 122 | 6-Methoxykaempferol 3,7-bis(3-acetylrhamnoside) | 710.23 | 1.92 | 1.16 | 3.08 | 2.04 |
| 123 | Kaempferol 3-(2''-p-coumaryl-rhamnoside)-7-rhamnoside | 723.19 | - | 8.52 | - | 0.30 |
| 124 | Kaempferol 3-(6G-malonylneohesperidoside) | 725.15 | 7.01 | 4.90 | 8.45 | 6.10 |
| 125 | Naringin 4'-glucoside | 725.23 | 1.54 | 2.22 | 0.78 | 0.49 |
| 126 | Kaempferide 3-rhamnoside-7-(6''-succinylglucoside) | 726.22 | 4.97 | 3.07 | 4.01 | 2.93 |
| 127 | Quercetin 3-(2'''-galloylglucosyl)-(1->2)-alpha-L-arabinofuranoside | 729.13 | 2.36 | 2.21 | 0.13 | 0.05 |
| 128 | Kaempferol 3-[6''-(3-hydroxy-3-methylglutaryl)glucoside]-7-glucoside | 735.18 | 1.24 | 1.11 | 3.70 | 3.75 |
| 129 | Prunin 4'',6''-di-O-gallate | 737.13 | 1.15 | 1.10 | 0.29 | 0.14 |
| 130 | 5,7-Dihydroxy-3,6,8,4'-tetramethoxyflavone 7-glucosyl-(1->3)-galactoside | 737.17 | 1.07 | - | 5.51 | - |
| 131 | Okanin 4'-O-(2''-O-caffeoyl-6''-O-p-coumaroylglucoside) | 739.16 | 1.03 | 1.78 | 5.01 | 15.10 |
| 132 | Kandelin A-1 | 739.17 | - | 1.68 | - | 3.06 |
| 133 | Kaempferol 3-(2''-rhamnosylrutinoside) | 741.22 | - | 2.93 | - | 0.35 |
| 134 | Quercetin 3-(2''-galoylrutinoside) | 743.14 | 2.90 | 2.59 | 0.10 | 0.06 |
| 135 | Delphinidin 3-robinobioside-5-glucoside | 754.20 | 2.03 | 1.65 | 0.46 | 0.48 |
| 136 | Quercetin 3-(2G-(E)-p-coumaroylrutinoside) | 755.18 | 1.43 | - | 4.68 | - |
| 137 | Quercetin 3-O-(6"-malonyl-glucoside) 7-O-glucoside | 757.14 | 2.58 | 1.96 | 89334.67 | 71231.67 |
| 138 | Chalconaringenin 2'-O-glucoside 4'-O-gentobioside | 757.22 | 1.20 | 1.05 | 8.30 | 8.41 |
| 139 | Quercetin 3-[rhamnosyl-(1->2)-rhamnosyl-(1->6)-glucoside] | 757.22 | - | 5.15 | - | 0.35 |
| 140 | Quercetin 3-(2''-p-hydroxybenzoyl-4''-p-coumarylrhamnoside) | 759.15 | 1.12 | 2.13 | 60.72 | 245.40 |
| 141 | Sagittatoside C | 763.24 | - | 1.36 | - | 0.34 |
| 142 | Isovitexin 4'-O-glucoside 2''-O-(E)-ferulate | 769.20 | - | 1.56 | - | 5.96 |
| 143 | Pollenitin 8-butyrate | 771.20 | - | 1.11 | - | 0.33 |
| 144 | Quercetin 3-rhamnosyl-(1->6)-glucosyl-(1->6)-galactoside | 773.21 | 3.05 | - | 2.36 | - |
| 145 | Delphinidin 3-lathyroside 5-(6-acetylglucoside) | 800.20 | 1.32 | 1.01 | 0.31 | 0.39 |
| 146 | Albanin G | 805.29 | - | 1.08 | - | 24.31 |
| 147 | Hexandraside D | 805.29 | 1.12 | 1.72 | 125.02 | 330.18 |
| 148 | Quercetin 3-(6'''-sinapylglucosyl)(1->2)-galactoside | 813.19 | 2.23 | - | 18.21 | - |
| 149 | Isorhamnetin 3-rutinoside 4'-rhamnoside | 815.22 | 2.57 | 2.17 | 3.68 | 3.54 |
| 150 | Kaempferol 3-(6'''-caffeylglucosyl)-(1->2)-galactoside | 817.18 | - | 1.18 | - | 0.23 |
| 151 | Gericudranins B | 819.20 | 1.47 | - | 2.85 | - |
| 152 | Acacetin 7-(4''''-Acetylrhamnosyl)-(1->6)-glucosyl-(1->3)-(6''-acetylglucoside) | 837.24 | - | 1.29 | - | 0.51 |
| 153 | Myricetin 3-(3'''-6'''-diacetylglucosyl)-(1->4)-(2'',3''-diacetylrhamnoside) | 839.19 | 1.59 | 1.00 | 2.35 | 1.79 |
| 154 | alpha-Rhamnorobin | 863.20 | 3.82 | 4.19 | 0.46 | 0.16 |
| 155 | Robinetinidol-(4alpha->8)-catechin-(6->4alpha)-robinetinidol | 865.20 | 1.46 | - | 3.47 | - |
| 156 | Epicatechin-(4beta->6)-epicatechin-(2beta->7,4beta->8)-epicatechin | 865.20 | 3.06 | 1.77 | 5.65 | 3.42 |
| 157 | Procyanidin C1 | 867.21 | 8.18 | 4.54 | 2.95 | 1.95 |
| 158 | 7-Chloro-3,3',4',5,6,8-hexamethoxyflavone | 871.18 | 1.24 | - | 39.03 | - |
| 159 | Munetone | 871.29 | - | 1.05 | - | 0.40 |
| 160 | Gallocatechin-(4alpha->8)-catechin-(4alpha->8)-catechin | 883.21 | 1.07 | - | 1.67 | - |
| 161 | Albanin H | 885.31 | - | 1.17 | - | 32.36 |
| 162 | Sissotrin | 891.23 | - | 1.21 | - | 3.87 |
| 163 | Kaempferol 3-(6'''-(E)-p-coumaroylglucosyl)-(1->2)-glucoside-7-rhamnoside | 901.24 | - | 1.01 | - | 0.23 |
| 164 | Quercetin 3-(6'''-p-coumarylglucosyl)(1->2)-rhamnoside 7-glucoside | 917.24 | - | 2.14 | - | 0.44 |
| 165 | Kaempferol 3-[2''-(6'''-coumaroylglucosyl)-rhamnoside] 7-glucoside | 947.24 | - | 2.71 | - | 0.30 |
| 166 | Quercetin 3-(6-[4-glucosyl-p-coumaryl]glucosyl)(1->2)-rhamnoside | 963.24 | - | 1.97 | - | 0.27 |
| 167 | Kaempferol 3-O-sinapoyl-sophoroside 7-O-glucoside | 977.25 | 3.13 | 4.75 | 8.35 | 27.20 |
| 168 | Kaempferol 3-neohesperidoside-7-(2''-ferulylglucoside) | 977.25 | 1.04 | 1.80 | 41.84 | 179.00 |
| 169 | 2',4',6',3,4-Pentahydroxy-3'-geranyl-5-prenyldihydrochalcone | 989.54 | - | 1.07 | - | 18.02 |
| **Organic acids (68)** | | | | | | |
| 1 | Glyoxylic acid | 72.99 | - | 1.19 | - | 0.50 |
| 2 | Malonic acid | 103.00 | 1.17 | 1.11 | 0.31 | 0.20 |
| 3 | L-Leucine | 130.09 | 1.36 | 1.24 | 0.15 | 0.03 |
| 4 | L-Asparagine | 131.05 | 1.19 | - | 0.03 | - |
| 5 | Malic acid | 133.01 | - | 1.09 | - | 0.66 |
| 6 | 2-Amino-4-ethoxy-3-hydroxybutanoic acid | 144.07 | 1.46 | 1.40 | 0.36 | 0.19 |
| 7 | Oxoglutaric acid | 145.01 | 1.98 | 1.80 | 0.51 | 0.41 |
| 8 | L-trans-5-Hydroxy-2-piperidinecarboxylic acid | 146.08 | 4.62 | 5.61 | 0.62 | 0.28 |
| 9 | D-Glutamate | 148.06 | 1.01 | 1.01 | 0.69 | 0.58 |
| 10 | Oxalosuccinic acid | 189.00 | 1.68 | 1.65 | 0.43 | 0.27 |
| 11 | 2-Keto-glutaramic acid | 190.04 | 1.18 | 1.16 | 0.38 | 0.25 |
| 12 | Citric acid | 191.02 | 3.33 | - | 0.67 | - |
| 13 | Isocitrate | 191.02 | - | 2.47 | - | 1.84 |
| 14 | Daucic acid | 203.02 | 2.87 | 2.00 | 4.74 | 3.40 |
| 15 | 3-Oxoadipic acid | 205.03 | - | 1.01 | - | 11.64 |
| 16 | Serinyl-Isoleucine | 219.13 | 2.06 | 1.77 | 0.06 | 0.02 |
| 17 | N-Undecanoylglycine | 226.18 | - | 1.02 | - | 0.45 |
| 18 | Isoleucylproline | 229.15 | 2.09 | 1.81 | 0.08 | 0.02 |
| 19 | Threoninyl-Isoleucine | 233.15 | 2.02 | 1.74 | 0.08 | 0.04 |
| 20 | Pyroglutamylvaline | 246.14 | 1.38 | 1.18 | 0.04 | 0.01 |
| 21 | L-beta-aspartyl-L-leucine | 247.13 | 1.26 | 1.08 | 0.04 | 0.01 |
| 22 | Tyrosyl-Alanine | 253.12 | 1.43 | 1.24 | 0.07 | 0.02 |
| 23 | Isoleucyl-Glutamine | 260.16 | 1.02 | - | 0.12 | - |
| 24 | Glutaminyl-Leucine | 260.16 | 2.16 | 1.87 | 0.06 | 0.01 |
| 25 | Isoleucyl-Glutamate | 261.14 | 1.38 | 1.21 | 0.11 | 0.03 |
| 26 | Valyl-Phenylalanine | 265.15 | 1.51 | 1.33 | 0.11 | 0.04 |
| 27 | Phenylalanyl-Valine | 265.15 | 1.18 | 1.14 | 0.22 | 0.04 |
| 28 | Phenylalanyl-Threonine | 267.13 | 1.01 | - | 0.05 | - |
| 29 | Histidinyl-Isoleucine | 269.16 | 1.27 | 1.08 | 0.06 | 0.03 |
| 30 | Elenaic acid | 287.08 | 1.45 | 1.68 | 2.30 | 2.99 |
| 31 | 2'-Deoxymugineic acid | 287.12 | 1.26 | - | 1.99 | - |
| 32 | N-lactoyl-Tryptophan | 294.14 | 1.54 | 1.32 | 0.04 | 0.02 |
| 33 | Hydroxyprolyl-Tyrosine | 295.13 | 1.74 | 1.51 | 0.08 | 0.03 |
| 34 | Isoleucyl-Tyrosine | 295.16 | 1.01 | - | 0.12 | - |
| 35 | Tyrosyl-Isoleucine | 295.16 | 1.49 | 1.31 | 0.08 | 0.01 |
| 36 | N-Jasmonoylisoleucine | 322.20 | 1.43 | 1.31 | 0.20 | 0.09 |
| 37 | Tyrosyl-Phenylalanine | 329.15 | 1.07 | 1.01 | 0.24 | 0.14 |
| 38 | Glutathione | 330.06 | - | 2.22 | - | 0.20 |
| 39 | Lysyl-Arginine | 341.17 | 1.06 | - | 3.26 | - |
| 40 | Na-p-Hydroxycoumaroyltryptophan | 349.12 | 1.30 | 2.44 | 128.09 | 501.88 |
| 41 | Dacarbazine | 365.19 | 1.83 | 1.28 | 16.82 | 12.73 |
| 42 | Enalaprilat | 366.20 | 1.14 | 1.07 | 0.20 | 0.05 |
| 43 | L-argininium(1+) | 389.20 | 1.14 | - | 0.08 | - |
| 44 | Acetyl tributyl citrate | 403.23 | 7.35 | 11.87 | 0.82 | 0.49 |
| 45 | Alanyl-Asparagine | 405.17 | - | 2.43 | - | 0.40 |
| 46 | (2R,2'S)-Isobuteine | 413.11 | 1.12 | - | 80.81 | - |
| 47 | 3-Methoxytyrosine | 421.16 | - | 1.01 | - | 3.00 |
| 48 | (S)-9-Hydroxy-10-undecenoic acid | 423.27 | 1.22 | 1.20 | 0.25 | 0.07 |
| 49 | Stearyl citrate | 427.30 | 1.29 | 1.21 | 0.25 | 0.10 |
| 50 | N-acetyl-L-2-aminoadipate(2-) | 429.15 | 1.56 | 1.37 | 0.23 | 0.21 |
| 51 | Alanyl-Glutamine | 433.21 | 1.11 | 1.61 | 3.46 | 7.91 |
| 52 | Folic acid | 459.18 | - | 4.30 | - | 0.18 |
| 53 | 3-Hydroxymonoethylglycinexylidide | 462.31 | 1.20 | 1.36 | 297.76 | 655.09 |
| 54 | Dynorphin B (10-13) | 468.28 | 1.21 | 1.16 | 4.84 | 5.01 |
| 55 | Cysteineglutathione disulfide | 471.09 | 1.42 | 1.49 | 0.29 | 0.07 |
| 56 | Na-Hexanoyl-Nb-inosityltryptophan | 487.21 | - | 1.63 | - | 0.36 |
| 57 | Histidinyl-Lysine | 565.32 | 1.13 | - | 10.84 | - |
| 58 | Desmosine | 571.28 | 2.35 | 2.34 | 3.29 | 5.44 |
| 59 | 6-Hydroxysandoricin | 585.24 | 1.03 | 2.12 | 6.76 | 33.11 |
| 60 | Methionyl-Phenylalanine | 591.23 | - | 1.15 | - | 0.41 |
| 61 | Vignatic acid A | 598.28 | 1.04 | - | 0.12 | - |
| 62 | Indoleacetyl glutamine | 605.24 | 3.72 | 4.20 | 0.62 | 0.46 |
| 63 | Oxidized glutathione | 611.14 | 1.79 | 2.02 | 0.45 | 0.21 |
| 64 | Bis-gamma-glutamylcysteinylbis-beta-alanine | 685.18 | - | 1.36 | - | 2.89 |
| 65 | Neocasomorphin | 717.38 | - | 1.15 | - | 317.17 |
| 66 | S-Lactoylglutathione | 757.20 | 1.14 | 1.02 | 0.18 | 0.11 |
| 67 | Hydroxy Ritonavir | 781.30 | - | 1.55 | - | 11.57 |
| 68 | Biocytin | 783.33 | 1.88 | 1.79 | 0.22 | 0.09 |
| **Terpenoids (35)** | | | | | | |
| 1 | beta-Phellandrene | 137.13 | 1.25 | - | 2.40 | - |
| 2 | trans-p-Menth-2-ene-1,4-diol | 153.13 | 1.47 | - | 14.91 | - |
| 3 | 5-Isopropyl-2-(2-methylpropyl)-2-cyclohexen-1-one | 177.16 | 1.07 | - | 4.14 | - |
| 4 | Monomenthyl succinate | 301.16 | 1.24 | 1.43 | 0.41 | 0.09 |
| 5 | Nepetaside | 329.16 | 4.11 | 6.67 | 0.82 | 0.51 |
| 6 | (1R,3S,4S,6R)-6,9-Dihydroxyfenchone 6-O-b-D-glucoside | 345.15 | - | 1.61 | - | 0.20 |
| 7 | (4S,6R)-p-Mentha-1,8-diene-6,7-diol 7-glucoside | 348.20 | 1.62 | 1.37 | 0.03 | 0.01 |
| 8 | (1S,2S,4R,5S,7S)-2,5,7-Fenchanetriol 2-O-b-D-glucoside | 366.21 | 1.18 | - | 16.56 | - |
| 9 | Monotropein | 371.10 | 1.59 | 1.86 | 2.85 | 4.86 |
| 10 | 6-Hydroxy-2-bornanone glucoside | 375.16 | 2.23 | 1.37 | 20.64 | 8.58 |
| 11 | Tsangane L 3-glucoside | 375.24 | 1.72 | 1.05 | 4.13 | 3.21 |
| 12 | Oleoside 11-methyl ester | 387.13 | 1.10 | - | 113.82 | - |
| 13 | Bakkenolide D | 389.14 | 1.77 | - | 0.35 | - |
| 14 | Veranisatin C | 390.14 | 1.58 | 1.47 | 4.58 | 5.65 |
| 15 | Sonchuionoside C | 404.23 | 1.63 | 1.67 | 2.90 | 3.63 |
| 16 | Citroside A | 404.23 | - | 1.01 | - | 5.18 |
| 17 | Oleoside dimethyl ester | 419.15 | - | 1.99 | - | 14.30 |
| 18 | Betulin | 425.38 | - | 1.01 | - | 9.74 |
| 19 | UVAOL | 425.38 | - | 2.12 | - | 74.21 |
| 20 | (4R,5S,7R,11x)-11,12-Dihydroxy-1(10)-spirovetiven-2-one 12-glucoside | 432.26 | 2.55 | 2.68 | 2.61 | 3.22 |
| 21 | Ganoderol A | 439.36 | 3.81 | 3.37 | 2.13 | 2.16 |
| 22 | Acuminoside | 466.26 | 2.29 | - | 1.60 | - |
| 23 | Gibberellin A1 glucosyl ester | 493.21 | 1.04 | - | 2.54 | - |
| 24 | Arjunolic acid | 506.38 | - | 1.09 | - | 6.61 |
| 25 | Hovenidulcigenin A | 562.37 | 1.59 | 1.70 | 0.35 | 0.11 |
| 26 | Ligustroside | 569.19 | 1.36 | 2.17 | 3.90 | 9.55 |
| 27 | 10-Acetoxyoleuropein | 597.18 | 2.31 | - | 1.37 | - |
| 28 | cis-p-Coumaroylcorosolic acid | 619.40 | - | 1.29 | - | 25.68 |
| 29 | 3-O-cis-Coumaroylmaslinic acid | 619.40 | - | 1.34 | - | 39.87 |
| 30 | Bisnorbadioquinone A | 649.06 | 1.88 | - | 1.42 | - |
| 31 | Lyciumoside IX | 715.35 | - | 1.16 | - | 881.44 |
| 32 | Dulcoside A | 833.38 | 1.81 | 2.25 | 81.20 | 133.97 |
| 33 | Stevioside | 849.37 | - | 1.18 | - | 134.47 |
| 34 | Kudzusaponin SA2 | 943.49 | - | 1.39 | - | 418.13 |
| 35 | Rebaudioside C | 995.43 | - | 1.18 | - | 1772.76 |
| **Tannins (2)** | | | | | | |
| 1 | beta-Glucogallin | 331.07 | 1.51 | - | 2.25 | - |
| 2 | 3,4-Hexahydroxydiphenoylarabinose | 451.05 | - | 1.68 | - | 0.43 |
